# Development and psychometric properties of the health-risk behavior inventory for Chinese adolescents

**DOI:** 10.1186/1471-2288-12-94

**Published:** 2012-07-07

**Authors:** Mengcheng Wang, Jinyao Yi, Lin Cai, Muli Hu, Xiongzhao Zhu, Shuqiao Yao, Randy P Auerbach

**Affiliations:** 1 Medical Psychological Institute, Second Xiangya Hospital, Central South University, #139 Ren-Min Zhong Road, Changsha 410011, China; 2 Child and Adolescent Mood Disorders Laboratory, McLean Hospital, Harvard Medical School, 115 Mill Street, Belmont, MA 02478, USA

**Keywords:** Adolescent, Health-Risk behavior, Reliability, Validity

## Abstract

**Background:**

There is a growing body of research investigating adolescent risk behaviors in China, however, a comprehensive measure that evaluates the full spectrum of relevant risk behaviors is lacking. In order to address this important gap, the current study sought to develop and validate a comprehensive tool: the Health-Risk Behavior Inventory for Chinese Adolescents (HBICA).

**Methods:**

Adolescents, ages 14–19 years (n = 6,633), were recruited from high schools across 10 cities in mainland China. In addition, a clinical sample, which included 326 adolescents meeting DSM-*IV* criteria for Conduct Disorder, was used to evaluate predictive validity of the HBICA. Psychometric properties including internal consistency (Cronbach’s alpha), test-retest reliability, convergent validity, and predictive validity were analyzed.

**Results:**

Based upon item analysis and exploratory factor analysis, we retained 33 items, and 5 factors explained 51.75% of the total variance: Suicide and Self-Injurious Behaviors (SS), Aggression and Violence (AV), Rule Breaking (RB), Substance Use (SU), and Unprotected Sex (US). Cronbach’s alphas were good, from 0.77 (RB) to 0.86 (US) for boys, and from 0.74 (SD) to 0.83(SS) for girls. The 8 weeks test–retest reliabilities were moderate, ranged from 0.66 (AV) to 0.76 (SD). External validities was strong, with Barratt Impulsiveness Scale-11 was 0.35 (*p* < 0.01), and with aggressive behavior and rule-breaking behavior subscales of the Youth Self Report were 0.54 (*p* < 0.01) and 0.68 (*p* < 0.01), respectively. Predictive validity analysis also provided enough discriminantity, which can distinguish high risky individual effectively (cohen’ *d* = 0.79 – 2.96).

**Conclusions:**

These results provide initial support for the reliability and validity of the Health-Risk Behavior Inventory for Chinese Adolescents (HBICA) as a comprehensive and developmentally appropriate assessment instrument for risk behaviors in Chinese adolescents.

## Background

During adolescence health-risk behaviors (HRBs) or/and problem behaviors increase the likelihood of negative long-term consequences (e.g., unintended pregnancy and sexually transmitted infections) and predict decreased psychosocial functioning throughout adulthood [1-3]. According to alarming national data from the United States, within 30 days prior to taking the Youth Risk Behavioral Survey (YRBS), high school students reported the following: 34.2% were sexually active, 38.9% of sexually active students had not used a condom during their last sexual intercourse, 41.8% drank alcohol, and 20.8% used marijuana [4]. Additionally, during a 12-month period, 31.5% had been in a physical fight and 6.3% of students had attempted suicide [4]. Notably, Chinese adolescents utilize HRBs less frequently as compared to Western youth [5-7]; however, such behaviors appear to be increasing in frequency among Chinese youth [8-10]. Presently, China has the world’s largest population, and it also includes the largest number of adolescents. Specifically, approximately 10.85% of the Chinese population is between the ages of 12 and 17 (n = 124,856,711)[11], and as rates of deviant behaviors are increasing in frequency among youth, it is important to develop an instrument, which will delineate the types and frequencies of HRBs that individuals utilize. In doing so, researchers and clinicians can then develop strategies to curb escalating engagement in HRBs.

While there is a growing body of research examining adolescent risk behaviors in China [5-10,12-14], there is not a comprehensive measure that was developed using an *emic* approach. Rather, researchers have more typically applied an *etic* method in which a measure was created in Western settings and then translated the instrument for use within mainland China. These initial steps provided a wealth of important information about patterns of behaviors among Chinese adolescents, and importantly, it also identified subtle differences, which would be critical to address in the development of a *native* tool.

To date, a number of screening instruments have been created to assess HRBs or problem-related behaviors [15-21], however, the scope of each of these measures is limited and cannot evaluate the full spectrum of HRBs. For example, the Adolescent Risk Behavior Screen (ARBS) [20] contains 9 items; however, five of these items assess substance use and the remaining 4 items each assess a specific risk behavior (e.g., fighting and unhealthy eating). Similarly, the Adolescent Risk Inventory (ARI) [21] fails to evaluate the full spectrum of health-risk behaviors as only three specific type of risk behaviors were encompassed.

Comparatively, the more comprehensive instruments assessing multiple domains of potential problems tend to be lengthy and time consuming, which may limit the clinical application. For example, the Problem Oriented Screening Instrument for Teenagers (POSIT) [22] and Child Behavior Checklist (CBCL) [23], both gold standards, are lengthy, 139 and 113 items, respectively. Alternatively, the Youth Risk Behavior Survey (YRBS) [24], which is administered to high school students in the United States every 2 years, measures risk behaviors across multiple domains, including substance use, sexual risk behavior, and violence. Although the YRBS has been shown to have good test-retest reliability [25], it is not easy to examine internal consistency coefficients in light of the dichotomous response scale (i.e., yes/no responses). Moreover, the data is not appropriate for further sophisticated statistical analyses (e.g., structure equation modeling), as most of the questions regarding specific behaviors only provide frequency data.

In order to address an important gap, the current study sought to develop and validate a comprehensive tool, which accounted for subtle behavioral differences that may emerge within adolescents from mainland China: the Health-Risk Behavior Inventory for Chinese Adolescents (HBICA). In doing so, we examined the psychometric properties including internal consistency (Cronbach’s alpha), test-retest reliability, convergent validity, and predictive validity of HBICA in a large and representative mainland China high school sample.

## Methods

### Participants

Participants in sample 1 were recruited from high schools in 10 urban areas across different parts of mainland China. These cities included Beijing, Shenyang, Langfang, Cangzhou, Hangzhou, Suzhou, and Guangzhou, Changsha and Yinchuan, and Chengdu. The final sample included 6,633 adolescents, and Table 1 includes demographic information pertaining to age, grade, gender, race, family background, and parental education status.

**Table 1:** Demographic Characteristics of the Community and Clinical Samples

**Demographic variables**	**Sample 1**				***p***	**Sample 2^a^**
	**Total (6,633)**	**Boys (3,280)**	**Girls (3,353)**	***χ*^*2*^**		**Total (326)**
Ethnicity				1.15	0.28	
Han nationality	94.5% (6,267)	94.8% (3,109)	94.2% (3,158)			94.2% (307)
Other ethnic minority	5.5% (366)	5.2% (171)	5.8% (195)			5.8% (19)
Grade				37.74	<0.01	
10th	44% (2,918)	43% (1,409)	45% (1,509)			
11th	31% (2,056)	34.3% (1,509)	27.8% (931)			
12th	25% (1,659)	22.7% (746)	27.2% (913)			
Only one child				68.99	<0.01	
Yes	65.7% (4,361)	70.6% (2,317)	61% (2,044)			48.8% (159)
No	34.3% (2,272)	29.4% (963)	39% (1,309)			51.2% (167)
Family composition				.57	0.90	
Nuclear families	91% (6,036)	91.2% (2,992)	90.8% (3,044)			77.9% (254)
Divorced families	4.1% (272)	3.9% (129)	4.3% (143)			11.3% (37)
Remarried families	2.7% (180)	2.7% (87)	2.8% (93)			6.8% (22)
Single-parent families	2.2% (145)	2.2% (72)	2.2% (73)			4% (13)
Paternal education				0.87	0.78	
Primary or less	51% (3,383)	51.6% (1,692)	50.4% (1,691)			55.8% (182)
High school	32.1% (2,129)	31.7% (1,040)	32.5% (1,089)			26.1% (85)
University	16.9% (1,121)	17.7% (581)	16.1% (540)			18.1% (59)
Maternal education				2.56	0.14	
Primary or less	59.4% (3,940)	58.3% (1,912)	60.5% (2,028)			62% (202)
High school	28.4% (1,884)	27.4% (899)	29.4% (985)			23% (75)
University	12.2% (809)	14.3% (469)	10.1% (340)			15% (49)

Note. a = 85.of the clinical sample was male, so the distribution of sample 2 not according to gender.

Additionally, a clinical population, sample 2, was recruited from the Medical Psychological Institute, Central South University, Second Xiangya Hospital, in Changsha, Hunan, People’s Republic of China. A total of 326 adolescents (ages = 13–17; M = 15.10 years, SD = 0.9, 85.5% male) met DSM-IV criteria for Conduct Disorder.

### Procedure and Data Collection

Prior to initiating the project, the Human Subjects Review Committee at Central South University provided approval of our study objectives and design. For adolescents participating in high schools across mainland China, parental consent was also required. Informed consent was given by parents and legal guardians, as well as by students, prior to the administration of self-report questionnaires. Participating adolescents were administered the HBICA and criterion measures including Barratt Impulsiveness Scale-11 version (BIS-11)[26] and Youth Self-Report Form (YSR)[23,27], during a predetermined 45-minute class period. Additionally, a total of 678 adolescents were invited to complete the HBICA 8 weeks after initial survey completion. These adolescents were chosen from participating schools in Suzhou and Yinchuan. This sample was representative of our population age (ages 14–19; M = 16.18, SD = 0.95) and gender (54% female and 46% male).

With respect to sample 2, all participants completed the self-report questionnaires as the same procedure as the sample 1. Additionally, the Structured Clinical Interview for DSM-IV (SCID) was used to generate current diagnoses for Conduct Disorder [28]. Two clinical psychology doctoral students conducted the interviews, and the Fleiss kappa was 0.74.

### Measures

*Health-Risk Behavior Inventory for Chinese Adolescents* (HBICA). HRB is a composite concept that includes a wide range of behaviors. Development of the HBICA was determined based on items included in the YRBS [4], which divides HRB into 6 categories: unintentional injuries and violence, tobacco use, alcohol and other drug use, sexual risk behaviors, unhealthy dietary behaviors, and physical inactivity, and the Risk Behavior Questionnaire for Adolescents (RBQ-A) [12-14], which classifies risk behavior into another six categories: unsafe sexual practices, aggressive and/or violent behaviors, rule breaking, dangerous, destructive, and/or illegal behaviors, suicide and self-injurious behaviors, and alcohol and/or drug use. Since the YRBS and RBQ-A contain overlapping assessment categories, we combined these sections into six domains in the HBICA: (1) Suicide and Self-Injurious Behaviors (SS), (2) Health-Compromising Behaviors (HCB), (3) Aggression and Violence (AV), (4) Rule Breaking (RB), (5) Substance Use (SU), and (6) Unprotected Sex (US). Based upon an extensive literature review and adaptations from related scales (i.e., YRBS and RBQ), we constructed a measure, which included a total of 50 items with 6–12 items per domain. An expert panel of 5 specialists in clinical psychology, health education, behavioral medicine, and pediatrics examined the initial questionnaire. The panel was asked to comment on each item regarding the accuracy, clarity, and relevance. Items were slightly modified based on expert reviews. Respondents reported the frequency of their engagement using a scale included in the RBQ-A: 0 = never, 1 = 1 time per month, 2 = 2–4 times per month, 3 = 2–3 times per week, and 4 = 4 times or more per week.

*The Chinese Version of the Barratt Impulsiveness Scale-11* (BIS-11). The BIS-11 is a widely used self-report questionnaire that asks participants to rate their frequency of common impulsive or non-impulsive activities on a 1 (rarely/never) to (almost always/always). The BIS-11 contains 30 items, and the sum of the ratings provides an overall impulsiveness score, with higher scores indicating greater levels of impulsivity. Past research has indicated that the instrument is reliable and valid to use among Chinese adolescents [26]. In the current study, the Cronbach’s alpha was .81, indicating high internal consistency.

*Youth Self-Report Form* (YSR) [23,27]. Two subscales from the YSR were included in the current study: Rule-Breaking Behavior (RB, 13 items) and Aggressive Behavior (AGG, 20 items). These questions are answered on a 3-point scale (0 = never true, 1 = sometimes true, 2 = always true). Excellent psychometric properties have been reported for the Chinese version, including strong test-retest reliability, inter-parent agreement, internal consistency, and high construct validity [23,27]. In the present study, the Cronbach’s alphas for RB and AGG were .71 and .68, respectively.

### Data analysis

Descriptive statistics, ANOVA, and exploratory factor analysis (EFA) were performed using SPSS version 18.0. Additionally, Mplus 5.1 was utilized in order to conduct confirmatory factor analyses (CFA).

Given the large sample size, two subsamples were generated using the “select case” function in SPSS, with random selection and sample size set at approximately 50%. The first subsample included 3,326 cases, and it was used for item analysis and EFA. The second subsample, with 3,307 cases, was used for Confirmatory Factor Analysis (CFA). When conducting EFA, factors were extracted by principle components and subjected to a varimax rotation. The scree plot test and Eigenvalues exceeding 1.0 were chosen to determine the number of factors. Items were retained based upon the following criteria: (1) the corrected item-total correlation was > .30 and statistically significant at *p* < .001, and (2) the factor loading was > .35 and there was no evidence of cross loading, loading at target factor was higher than that at nontarget factor.

Next, the retained items were subjected to CFA using the subsample. Since item distribution was non-normal, we used a robust maximum likelihood estimator for non-normality [29] to avoid potential data distortion. As part of this accepted practice [30], we evaluated the fit of the CFA model using Satorra-Bentler chi-square, root-mean-square error of approximation (RMSEA < 0.08), Tucker–Lewis index (TLI > 0.90), and a comparative fit index (CFI > 0.90).

Cronbach’s alpha coefficient, test-retest correlation, and corrected item-total correlation were all chosen to evaluate HBICA reliability. Pearson product–moment correlations determined scale concurrent validity, as well as independent sample *t*-tests and effect size (Cohen’s *d*) were chosen to evaluate predictive validity of the inventory. Two-way analysis of variance was used to compare scores across gender and grade.

## Results

### HBICA Item Analysis and Modifications

We used the corrected item-total correlation to retain an item. We calculated the corrected item-total correlations, and a question was removed if the item-total correlation was less than 0.30. Consequently, 8 items were removed: “Have you used illegal drugs?”, “Have you sold illegal drugs?”, “Have you had unclear propose drugs?”, “Have you ever engaged in sexual actives by force?”, and “Have you committed arson?”, “Have you been unfaithful to your boyfriend or girlfriend”, “Have you vomited or used laxatives to control your weight”, and “Have you starved yourself for weight control.”

In addition, all five questions regarding Health-Compromising Behaviors (HCB) were removed due to low item-total correlations. Those items included: “Do you have breakfast everyday?”, “Do you drink milk?”, “Do you drink soya-bean milk?”, “Do you drink soda/cola?”, and “Do you drink juice?”.

### Exploratory and Confirmatory Factor Analysis

Based on the item deletions specified above, EFA examined the associations among the remaining 37 items. The results from EFA revealed that five factors were extracted reasonably. Each factor contained 5 to 12 items. Items were deleted while cross-loaded on multiple factors (factor loading more than or equal to |0.35| on two or more factors) and/or did not load significantly on any factor (loading less than |0.30| on all factors). After removing the nonloading and cross-loading questions, 33 items remained. A new EFA was then conducted on these questions, and the analyses confirmed the 5-factor solution, which explained 51.75% of the total variance.

Using the other subsample (n = 3,307), we conducted a CFA to examine the 5-factor structure generated by the EFA described above. The fit indices included: NNFI = 0.90, CFI = 0.92, and RMSEA (90% CI) = 0.029 (0.029, 0.031). Such findings indicate that a 5-factor structure is an acceptable fit of the data. Factor loadings for all items were statistically significant and ranged from 0.42 to0 .87 (*p* < 0.01) (see Table 2).

**Table 2:** **Item-Total Correlation (*n*** **= 3307) and Standard Factor Loadings for the HBICA (*n*** **= 3,326)**

****Item content****	***Cr***	**AV**	**SS**	**US**	**SU**	**RB**
Verbal attack someone	0.42**	0.45				
Damaging others’ property	0.42**	0.44				
Making fun of someone for their appearance or physical defect	0.45**	0.45				
Bullying, threatening, or intimidating someone	0.55**	0.53				
Have been in a physical fight	0.63**	0.75				
Hitting, crushing, pushing, kicking or confining another	0.61**	0.62				
Ignore driving consequences	0.44**	0.44				
Revenge on someone	0.61**	0.61				
Blackmailed someone for money	0.49**	0.47				
Carried a weapon	0.59**	0.69				
Attempted to cut or scald self	0.60**		0.58			
Ideas of Suicide	0.69**		0.55			
Hurt self by biting, scratching, or knocking	0.58**		0.61			
Attempts at Suicide	0.72**		0.72			
Attempted suicide	0.62**		0.61			
Ever had sexual intercourse	0.50**			0.51		
Had sexual intercourse with at least one person	0.65**			0.71		
Drank alcohol or used drugs before sexual intercourse	0.61**			0.73		
Caused girl pregnancy (boy) or caused yourself pregnancy (girl)	0.57**			0.81		
Had sexual intercourse with stranger	0.55**			0.87		
Have you used cigarettes	0.68**				0.77	
Smoking under the pressure of companion	0.57**				0.58	
Binge Drinking	0.67**				0.73	
Irritable, lose temper, headache, or insomnia due to quit smoking	0.70**				0.67	
Drink alcohol out of control at a party	0.64**				0.43	
Drink alcohol to save face	0.68**				0.53	
Skip classes	0.60**					0.69
Run away from home	0.55**					0.64
Received warning, record a demerit, or punishment from school	0.57**					0.60
Cheat or plagiarize during examination	0.49**					0.48
Lie to family member	0.44**					0.42
Gambling	0.46**					0.54
Steal money from home	0.44**					0.43

*Note*: Cr = Corrected correlation coefficients; **p < 0.01 (2-tailed).

AV = Aggression and Violence, RB = Rule Breaking, US = Unprotected Sex, SS = Suicide and Self-Injurious and SU = Substance Use.

### Reliability

When examining the reliability of the HBICA, we conducted analyses in the full sample (i.e., sample 1). Cronbach’s alpha coefficients for the HBICA total scale and subscales are included in Table 3. In general, the internal consistency for the total instrument was comparable in boys (0.92) and girls (0.89), indicating strong internal consistency. Additionally, Pearson product–moment correlation coefficients for the 8-week interval examining test-retest reliability in a subset of the sample (n = 678) were moderate, ranging from 0.62 to 0 .76 (see Table 3).

**Table 3:** **Subscale Correlations of HBICA and Reliability Coefficients**^**a**^

**Scale**	**Cronbach’s Alpha**		**Test-retest reliability**	**BIS-Total**	**YRS-AGG**	**YRS-RB**	**(1)**	**(2)**	**(3)**	**(4)**	**(5)**
	**Boys**	**Girls**									
(1) AV	0.82	0.75	0.66**	0.33**	0.55**	0.61**	1				
(2) RB	0.77	0.75	0.70**	0.31**	0.45**	0.63**	0.73**	1			
(3) US	0.86	0.77	0.62**	0.12**	0.24**	0.38**	0.48**	0.47**	1		
(4) SS	0.79	0.83	0.73**	0.23**	0.38**	0.41**	0.41**	0.39**	0.38**	1	
(5) SU	0.81	0.74	0.76**	0.23**	0.39**	0.56**	0.68**	0.67**	0.40**	0.42**	1
(6) HBICA Total	0.92	0.89	0.76**	0.35**	0.54**	0.68**	0.77**^b^	0.77**^b^	0.51**^b^	0.45**^b^	0.72**^b^

*Note*: AV = Aggression and Violence, RB = Rule Breaking, US = Unprotected Sex, SS = Suicide and Self-Injurious, SU = Substance Use, HBICA = Health-Risk Behavior Inventory for Chinese Adolescents, BIS-Total = The total score of the Barratt Impulsiveness Scale-11, YRS-AGG = Aggressive Behavior subscale of the Youth Self-Report Form, and YRS-RB = Rule-Breaking Behavior subscale of the Youth Self-Report Form.

a = The analyses in full sample (sample 1) except test-retest reliability(*n* = 678); b. Corrected correlation coefficients for full sample

**p < .01 (2-tailed).

### Convergent and Predictive Validity

Table 3 provides correlations among HBICA subscales. All the correlation coefficients exhibit weak to high effect sizes (*r* = .38 – .73). The largest effect size was between AV and RB (*r* = .73), suggesting that these subscales represent interrelated, yet distinct, aspects of health risk behaviors. Additionally, correlations between the HBICA total/subscales and (a) BIS-11 (*r* = 0.35), (b) YSR-AGG (*r* = 0.24 - 0.55), and (c) YSR-RB (*r* = 0.38 - 0.68) are included in Table 3.

To examine whether the HBICA could effectively discriminate between behaviors from high-risk individuals and those in a community sample, we compared responses from high school students and clinical subjects diagnosed with conduct disorder. Results indicated that clinical subjects scored significantly higher scores as compared to a non-clinical community sample (see Table 4).

**Table 4:** **Comparison Scores of HBICA and Subscales between Non-Clinical (*n*** **= 6,633) and Clinical (*n*** **= 326) Sample**

**Scale**	**Student sample *M* ± *SD***	**Clinical sample *M* ± *SD***	***t***	**Cohen *d***
(1) AV	4.47 ± 3.99	12.65 ± 7.74	34.18**	1.93
(2) RB	3.17 ± 2.66	11.72 ± 5.80	52.16**	2.96
(3) US	0.25 ± 1.06	2.03 ± 3.09	25.58**	1.45
(4) SS	1.43 ± 2.63	3.59 ± 4.24	13.94**	0.79
(5) SU	1.86 ± 3.12	9.28 ± 5.98	39.45**	2.24
(6) HBICA Total	11.18 ± 9.84	39.27 ± 20.54	46.75**	2.65

*Note*: AV = Aggression and Violence, RB = Rule Breaking, US = Unprotected Sex, SS = Suicide and Self-Injurious,

SU = Substance Use, HBICA = Health-Risk Behavior Inventory for Chinese Adolescents.

**p < .01 (2-tailed).

### Gender and Grade Differences

Two-way ANOVAs were conducted for HBICA total and subscale scores, with grade and gender as independent variables. Table 5 shows means and standard deviations for HBICA responses. Our results showed significant interactions between gender and grade for HBICA total and all subscale scores with the exception of the SS subscale.

**Table 5:** Means and Standard Deviations for the HBICA as a Function of Gender and Grade

	**Grade 10**				**Grade 11**				**Grade 12**				**TOTAL**			
	**boys**		**girls**		**boys**		**girls**		**boys**		**girls**		**boys**		**girls**	
**Scale**	***M***	***SD***	***M***	***SD***	***M***	***SD***	***M***	***SD***	***M***	***SD***	***M***	***SD***	***M***	***SD***	***M***	***SD***
(1) AV	5.32	4.11	3.43	2.76	5.93	4.56	3.54	2.88	5.22	3.93	3.22	2.48	5.51	4.24	3.41	2.72
(2) RB	3.69	2.85	2.86	1.99	4.38	3.27	2.99	2.25	3.91	2.84	2.82	1.99	3.98	3.01	2.89	2.07
(3) US	0.33	1.50	0.06	0.51	0.57	1.98	0.14	0.79	0.28	1.38	0.04	0.29	0.40	1.66	0.08	0.56
(4) SS	1.19	2.24	1.45	2.45	1.34	2.45	1.52	2.45	1.08	2.06	1.18	2.08	1.22	2.28	1.40	2.36
(5) SU	2.41	3.32	1.11	2.04	3.37	3.94	1.22	2.08	2.82	3.51	1.10	1.89	2.83	3.61	1.14	2.01
(6) HBICA Total	12.75	10.76	8.88	7.40	15.43	12.31	9.37	7.82	13.18	10.77	8.29	6.39	13.77	11.38	8.86	7.28

*Note*: AV = Aggression and Violence, RB = Rule Breaking, US = Unprotected Sex, SS = Suicide and Self-Injurious, SU = Substance Use, HBICA = Health-Risk Behavior Inventory for Chinese Adolescents.

## Discussion

The current study sought to develop the Health-Risk Behavior Inventory for Chinese Adolescents (HBICA), which would provide a comprehensive measure for evaluating risk behaviors. Importantly, we examined the factor structure and psychometric properties in a robust sample of non-clinical and clinical adolescents, and the results strongly suggest that the instrument is a valid self-report measure for assessing multiple health-risk behaviors among Chinese adolescents.

In general, an ideal measure of adolescent health-risk behaviors explores a wide range of behaviors. In the current study, we proposed 6 categories; however, EFA and CFA supported 5-factor structure. Specifically, items from the HCB were excluded as a result of low associations among the items and low item-total correlations; however, several other studies have also found this effect [31,32]. For example, in a study with 1,782 high school adolescents, Turbin and colleagues [32] found weak associations (*r* ≤ 0.20) among the following four HCB indicators: unhealthy dietary habits, sedentary behavior, unsafe behavior, and poor dental hygiene. Similarly, Adams and colleagues [15] reported weak associations (*r* ≤ 0.20) with exercise as it related to both sexual activity and substance use. Taken together, these results support our findings and suggest that the HCB is a distinct type of that may not be best captured in an instrument examining problem behaviors.

It should be noted that in our 5-factor solution, items pertaining to alcohol use and tobacco use were collapsed into a single factor. While this result is consistent with some researchers [12-15], others have reported that they are distinct dimensions [33]. It is possible that this discrepancy may be explained by sample characteristics and cultural backgrounds. For example, smoking and drinking are symbolized as “showing off” behaviors, particularly in Chinese boys. Therefore, it is not surprising that smoking is highly associated with drinking behavior in Chinese adolescents [7].

With regard to survey reliability, Cronbach’s alpha coefficients for HBICA total score and its subscales suggested satisfactory internal consistency. Moreover, the results from an 8-week test–retest demonstrated reliability and internal-consistency, which indicated that the test–retest reliability of all subscales and total scale were satisfactory. These results indicated that the HBICA is a reliable tool for investigating multiple health-risk behaviors among Chinese adolescents.

Importantly, the intercorrelations among HBICA subscale scores had large effect sizes and were similar to those found in previous studies [33,34]. Our findings provide additional evidence that the subscales represent interrelated, but distinct, aspects of health-risk behavior. The observed relationships between the HBICA and other self-report measures also support the construct validity of our inventory. For example, our results revealed significant correlations between AV and RB subscales and the YSR-RB subscale. In addition, a moderate to high statistically significant positive relationship was found between the HBICA subscales and the subscales of the YSR, although the relationship was somewhat stronger for the YSR-RB subscale than for the YSR-AGG subscale. It should also be noted that the correlation between the AV subscale and the YSR-RB subscale was larger than with the YSR-AGG subscale. Moreover, this pattern was similar for all the HBICA subscales. Regardless, of correlation size, these results are consistent with previous research [35,36] and suggests that the HBICA possesses strong convergent and discriminant validity.

Critically, clinical participants with a diagnosis of conduct disorder reported significantly higher on the HBICA and its subscales as compared to the non-clinical community sample. This finding demonstrates that the HBICA survey effectively differentiated “high-risk” versus “normal” adolescents. Additionally, the discriminating effects across the 5 subscales of the HBICA were heterogeneous.

With respect to sex differences, boys reported engaging in higher levels of health-risk behavior than girls (except for SS). This finding is consistent with meta-analyses showing large effect sizes regarding gender differences (*d* > 0.20) [32,37] and similar results obtained with Chinese adolescents [5-8,14]. Additionally, education level also influenced HBICA scores. Grade 12 respondents reported lower scores on the total and subscale items compared to Grade 11 subjects. Similarly, Grade 10 respondents reported lower scores than Grade 11 participants. It should be noted that Grade 10 represents the beginning of high school in China, or a participant’s freshman status. Therefore, these findings may be explained by the sharp increase in academic pressure following middle school completion and the continual increase in pressure for high school students to gain entrance into a better college [38].

There are several limitations to this study. First, while the HBICA is an advancement over other instruments used among Chinese adolescents, it is a self-report measure, and thus, it is subject to response bias. Future investigations would benefit from using a multi-informant approach including assessments from parents, teachers, and peers. Second, this study only examined the HBICA in a sample of urban adolescents, further studies should include rural sample to support the robustness of the HBICA.

## Conclusions

This study developed and validated the psychometric properties of a new comprehensive measure: the Health-Risk Behavior Inventory for Chinese Adolescents (HBICA). The study identified 5 factors to assess health-risk behaviors: Suicide and Self-Injurious Behavior (SS), Aggression and Violence (AV), Rule Breaking (RB), Substance Use (SU), and Unprotected Sex (US), and these subscales exhibited internal consistency and reliability. Moreover, our results demonstrated that the HBICA was an effective tool for distinguishing responses from Conduct Disorder adolescents and a non-clincial sample of adolescents. Moving forward, research in mainland China will benefit from the creation and utilization of an instrument, which specifically assesses patterns of behaviors affecting Chinese youth.

CBCL: Child Behavior Checklist; YSR: Youth Self Report; BIS-11: Barratt Impulsiveness Scale-11 version; ARI: Adolescent Risk Inventory; RBQ-A: Risk Behavior Questionnaire for Adolescent; EFA: Exploratory Factor Analysis; CFA: Confirmatory Factor Analysis; TLI: Tucker–Lewis Index; CFI: Comparative Fit Index; RMSEA: Root-Mean-Square Error of Approximation; SCID: Structured Clinical Interview

## Abbreviations

HBICA, Health-risk behavior inventory for Chinese adolescent; HRB, Health-risk behavior; SS, Suicide and self-injurious; HCB, Health-compromising behaviors; AV, Aggression and violence; RB, Rule breaking; SU, Substance use; US, Unprotected sex; ARBI, Adolescent risky behavior inventory; YRBS, Youth risk behavior survey.

## Competing interests

The authors declare that they have no conflicts of interests.

## Authors’ contributions

SY, XZ and JY were involved in the design of the study. MW, LC and MH was responsible for data gathering. MW analyzed the data. MW and RPA wrote the manuscript. All authors have read and approved the final version of the manuscript.

## Pre-publication history

The pre-publication history for this paper can be accessed here:

http://www.biomedcentral.com/1471-2288/12/94/prepub
